# Current Trends in Applications of Circulatory Microchimerism Detection in Transplantation

**DOI:** 10.3390/ijms20184450

**Published:** 2019-09-10

**Authors:** Hajnalka Andrikovics, Zoltán Őrfi, Nóra Meggyesi, András Bors, Lívia Varga, Petra Kövy, Zsófia Vilimszky, Fanni Kolics, László Gopcsa, Péter Reményi, Attila Tordai

**Affiliations:** 1 Laboratory of Molecular Genetics, Central Hospital of Southern Pest National Institute of Hematology and Infectious Diseases, 1097 Budapest, Hungary; 2 Department of Pathophysiology, Semmelweis University, 1089 Budapest, Hungary; 3 School of PhD Studies, Semmelweis University, 1085 Budapest, Hungary; 4 Hungarian National Blood Transfusion Service, 1113 Budapest, Hungary; 5 Department of Hematology and Stem Cell Transplantation, Central Hospital of Southern Pest National Institute of Hematology and Infectious Diseases, 1097 Budapest, Hungary; 6 Department of Transfusion Medicine, Semmelweis University, 1089 Budapest, Hungary

**Keywords:** microchimerism, solid organ transplantation, hematopoietic stem cell transplantation, genetic marker, single nucleotide polymorphism, deletion/insertion polymorphism

## Abstract

Primarily due to recent advances of detection techniques, microchimerism (the proportion of minor variant population is below 1%) has recently gained increasing attention in the field of transplantation. Availability of polymorphic markers, such as deletion insertion or single nucleotide polymorphisms along with a vast array of high sensitivity detection techniques, allow the accurate detection of small quantities of donor- or recipient-related materials. This diagnostic information can improve monitoring of allograft injuries in solid organ transplantations (SOT) as well as facilitate early detection of relapse in allogeneic hematopoietic stem cell transplantation (allo-HSCT). In the present review, genetic marker and detection platform options applicable for microchimerism detection are discussed. Furthermore, current results of relevant clinical studies in the context of microchimerism and SOT or allo-HSCT respectively are also summarized.

## 1. Introduction

Co-existence of genetically distinct/discordant tissues or cells is termed chimerism after the well-known Greek fusion creature. Based on the extent of the variant population, microchimerism is usually mentioned as a subgroup of chimerism in which the proportion of foreign cells or tissue does not exceed 1%. This proportion can readily be estimated in the circulation while quantitative comparison of major and minor populations is more challenging in cases of scattered cells or transplanted tissue.

Based on origin, naturally occurring and therapy related microchimerism can be distinguished. Naturally occurring microchimerism refers to the bidirectional interaction between the genetically distinct mother and fetus(es) resulting in the long-lasting presence of small numbers (well below 1%) of fetal cells in the mother and vice versa. The interesting immunological consequences of these coexistences have recently been elegantly reviewed [1] and is not discussed in the present review.

The term therapy related microchimerism describes situations primarily related to transplantations and occasionally transfusions. Subsequent to solid organ transplantations (SOT) a well-defined organ coexists with the recipient which rarely involves mixture of cells. In contrast, variable recipient–donor cell ratios are characteristic for patients treated by allogeneic hematopoietic stem cell transplantation (allo-HSCT).

The quantitative detection of chimerism in the circulation might serve as a biomarker with clinical relevance, which is based on polymorphic genetic structures (markers) similarly to those applied in forensic medicine for person identification. Large scale population studies such as the 1000 Genome Project characterized and classified millions of human variants in different populations allowing the identification of appropriate markers with high heterozygosity rate and discriminatory power. In the last decade, the development of highly sensitive, quantitative and accurate methods, e.g., digital PCR or next generation sequencing (NGS) enabled the precise quantitation of unprecedently low level molecular variants. As a result, the clinical application of these innovative technologies for microchimerism detection has started to enter the daily practice. In general, the paradigm of marker selection in transplantation dictates an allele choice as a marker that is not present in the dominant cell or molecule population: after SOT the donor should be marker positive and the recipient negative, while after allo-HSCT the opposite relationship is required.

Chimerism could be investigated on cellular or on cfDNA level. The latter consists mainly of small sized (around 150 base-pairs) double-stranded DNA fragments resulting from apoptosis, necrosis, immune-mediated cell damage, or release of nuclear DNA into the circulation predominantly from hematopoietic cells [2]. In the circulation, these fragments have a short half-life of approximately 1.5 h due to rapid hepatic and renal clearance [3].

In the present paper, we provide an extensive review of the available information about microchimerism in the context of various forms of transplantation and transplantation-related advanced therapies with a special emphasis on the available markers and the multitude of detection platforms in this high sensitivity range.

## 2. Polymorphic Genetic Markers Applicable for Chimerism Detection

### 2.1. Short Tandem Repeats (STR)

So far, more than 700,000 STRs (short tandem repeats) are characterized in human genome, for the most part they are considered as ‘junk DNA’, however some of them are located in protein coding region and associated with genetic disorders [4,5,6]. STRs—also referred to as microsatellites—consist of 2 to 6 nucleotide tandem repeats distributed throughout the genome. These loci are highly polymorphic and different alleles are determined by the varying number of nucleotide repeats (3 to 51 repeats). Short tandem repeat (STR) analysis is a standard and approved method applied in forensics and in hematopoietic chimerism quantification after allo-HSCT [7]. Nearly 100% informativity can be reached with a panel of 12–16 STR markers (Core STR Loci), including loci on sex chromosomes, that allow differentiation between individuals [8,9,10]. The major advantage of STRs is its multiallelic (up to 16 distinct variants) nature and consequential high informativity rate, which allows its application in all donor–recipient pairs or furthermore in transplants with multiple donors.

### 2.2. Single Nucleotide Polymorphism (SNP)

These represent the most frequently observed variations in the human genome, around 1/1000 basepairs [11]. In an average individual there are ten times more SNPs to be found compared to deletion insertion polymorphisms (DIP). Since the discrimination power of biallelic variants is lower compared to STRs, more loci have to be simultaneously tested to reach an acceptable informativity range. Reportedly, already seven SNP markers can be highly discriminative (97% informativity) in HLA-identical sibling pairs [12]. On the other hand, a study using a combined panel of markers described only 80% overall informativity with six SNPs, two null alleles (SRY, RhD) and two DIPs [13]. Almeida et al. calculated 81% informativity for eight biallelic SNP markers investigating 88 patient/donor pairs [14]. Fredriksson et al. used an array consisting of 51 SNPs to reach 100% informativity [15]. In the context of SOT, SNP assays have widely been used to detect donor-derived cfDNA (dd-cfDNA) amount in plasma with an early, method-focused application using digital PCR (see Section 3) in small sets of liver, kidney, and heart transplanted patients [16]. This approach was further developed and characterized in detail by Grskovic et al. [17] in the form of a commercial assay (CareDx, Brisbane, CA, USA) employing 266 SNPs and NGS (see Section 3) with a convincing validation in a large group of heart transplanted patients. The technical advances made possible a highly sophisticated approach of massive multiplexing allowing the utilization of more than 13,000 SNP markers [18], as well as an alternative commercial, NGS-based assay [19]. The enormous amount of information obtained from massively parallel SNP-typing by NGS also allows donor–recipient distinction in the absence of pre-testing potentially further simplifying the routine application [20,21].

### 2.3. Deletion Insertion Polymorphisms (DIP)

DIPs or INDELs are relatively short (up to about 50 bases in length), frequently biallelic variations located in non-coding regions of the genome displaying a lower mutation rate compared to STRs. As a consequence of the emergence of NGS technology and advanced software algorithms, the number of identified and characterized DIPs is rapidly increasing [22,23,24]. While searching the world’s largest public human variation database (dbSNP Build 152) and applying a filter for intergenic human DIPs, not restrictive to the length of the polymorphism, nearly 9 million hits are found. Biallelic DIPs are suitable and reliable markers which are now commonly used for monitoring donor–recipient tissue proportions in transplantation as well in person-identification in forensic medicine [25,26]. To monitor chimerism after allo-HSCT, Alizadeh et al. utilized 19 specific markers located on 9 different chromosomes reaching an informativity level of approximately 90%. This approach has been later validated by others [27,28,29]. A further study extended this system with two additional DIP markers and optimized their assay for SYBR green with a possible discrimination of 94% [30]. Subsequently, Pereira et al. developed a DIP-based multiplex assay for human identification, which uses 38 biallelic markers characterized by high heterozygosity rates in distinct population groups, reaching an approximate informativity of 100% [26]. As an alternative to hybridization probes, Goh et al. analyzed dd-cfDNA after liver transplantation by targeting DIPs by the amplification primers themselves [31]. The successful application of as few as 10 DIPs along with digital PCR for the detection of dd-cfDNA in a small cohort of heart transplanted patients published as a less resource demanding approach [32]. Besides these publicly available marker collections, a large selection of commercial kits are also available, indicating the importance of the approach [33,34,35,36,37]. Combinations of DIPs and SNPs (a total of 26 markers) were also tested with a genotype identification rate of 97% [38] and with another set (a total of 29 markers) resulting an informativity of 97% [39]. The major advantage of DIPs is the potential to use these markers in high sensitivity assay such as quantitative PCR or digital PCR (see Section 3) to reliably detect microchimerism equally important in SOT and allo-HSCT.

### 2.4. Other Special Markers Used in Monitoring

Evidently, application of sex determining markers (e.g., SRY, AMELY, DFFRY, SMCY) are only useful and informative in sex-mismatched transplanted patients both in allo-HSCT and SOT. The amelogenin gene has an X and Y linked variant (AMELY and AMELX). The basis of variability in this case is a 6-basepair-long deletion segment found in X chromosome (AMELX). Utilization of Y specific markers, e.g., SRY, rely on the detection of a gene exclusively located on chromosome Y. Both sex-specific markers are informative in allo-HSCT when the recipient is a male and the donor is a female [2,40,41], while in SOT the expected situation is exactly the opposite. Similarly, RhD blood group (which is a 58 kilobase-long DIP, i.e., RhD-negativity is equal to a complete deletion) is also a straightforward choice in monitoring RhD positive allo-HSCT patients with RhD negative donor. The opposite is applicable in SOT (an RhD negative recipient with an RhD positive donor) [13,42]. Besides these, another informative marker set is the HLA which information for both donor and recipient is readily available prior to transplantation. Disparate HLA-markers were successfully used to detect elevations in dd-cfDNA plasma concentrations associated with lung transplant complications. However, the major obstacle of the widespread use of this approach may be the need for the practically individual detection probe design depending on the actual HLA-disparities between the donor and recipient [43].

### 2.5. Guidelines for Marker Selection to Detect Clinical Chimerism

An ideal marker should be located in non-coding region ruling out the chance it is linked to genetic diseases, plays a role as genetic susceptibility factor or influences certain disease progression or response to therapy. It should be biallelic, differing by at least 2 basepairs, with a minimum allele frequency of at least 0.25 and a high level of heterozygosity (>0.4). Their chromosomal localization should be as distant as possible or on different chromosomes and they should also be located in sufficient distance from repetitive regions [25,26]. When designing SNPs as markers, loci with C/T and G/A polymorphisms are preferred to achieve more specific allele discrimination [38]. The recommended number of markers allowing sufficient informativity (>95%) for STR varies between 12 and 16, while for DIPs and SNPs a larger set is required (up to 52 markers). For follow-up diagnostics, more than one marker is recommended to avoid false negative results e.g., due to allele-dropout during the polymerase chain reaction (PCR) or due to the deletion of chromosomal region of the chosen marker in hematological malignancies [9,37]. Loss of chromosome Y was observed not only in hematological malignancies, but also in aging healthy males.

## 3. Comparison of Microchimerism Detection Techniques

As allo-HSCT became widespread, an imperative need arose to repeatedly discriminate recipient and donor cells in the circulation after allo-HSCT for which various techniques have been developed. Initially, these approaches had limited sensitivity in the range of 10% recipient cells allowing only to characterize chimerism and not microchimerism. The most commonly used chimerism-detection methods in this time period were XY fluorescence in-situ hybridization and polymerase chain reaction (PCR) based amplification of variable number of tandem repeats (VNTR) or short tandem repeats (STR). The PCR-based technologies more recently predominantly use fragment analyses (FA) making it possible reach a sensitivity range of 1% still unsuitable for the reliable detection of microchimerism (see the detailed review of Clark et al. [9]). Systematic comparison of three most widely used microchimerism detection technologies with reference to the gold-standard chimerism detection technique, fragment analysis is shown in Table 1.

### 3.1. Real-Time Quantitative PCR (qPCR)

To increase the sensitivity of chimerism monitoring in transplanted patients, several studies proposed real-time quantitative PCR (qPCR) method to detect single nucleotide polymorphisms (SNPs) or short deletions/insertions (DIP). QPCR allows 0.1% sensitivity quantification of the minor genotype [12,25,28,37,38,44]. The vast majority of studies employ TaqMan technology requiring a hybridization probe labeled with two different fluorescent dyes: a reporter (FAM), and a quencher (TAMRA). When the probe is intact, fluorescent energy transfer occurs and the reporter fluorescence is readily absorbed by the quencher. In case of precise hybridization of the probe to its target, during the extension phase of the PCR cycle, the 5’-3’ exonuclease activity of the DNA-polymerase cleaves the TaqMan probe and releases the reporter dye, resulting in an increase of the reporter dye fluorescent signal [45]. Probes can be labeled with alternative, distinguishable reporter dyes allowing duplexing in a single reaction. The disadvantage of the qPCR technique is the requirement of labor-intensive optimization and the need for replicates (duplicates, triplicates) in each run for each target to provide the most accurate result [12,25].

### 3.2. Digital PCR (dPCR)

Digital PCR has initially been used to quantify low-copy number fetal DNA in maternal plasma [46]. This innovative approach is based on partitioning of the PCR in multiple nanoliter chambers or droplets, and after the amplification, each chamber/droplet is counted positive or negative for a specific polymorphism [47]. With increasing the number of partitions (e.g., performing replicates), sensitivity of the test can be improved. A further innovation of this technique, droplet digital PCR (ddPCR), is easily automated and does not require a dedicated PCR machine. In contrast to qPCR which allows a real time approach, dPCR is an end-point assay with the determination of the positive droplet fraction and Poisson statistics calculating the absolute number of starting copies making calibration curves unnecessary [47,48]. Due to the simultaneous presence of reference amplification in each reaction during dPCR, as well as a consequence of unprecedented precision, replicates are not needed in cases with target concentrations above the limit of detection. This method is less sensitive to inhibitors than fragment analysis or qPCR. The technique is highly suitable for the detection of rare events in the presence of high background. Performing the quality control test on artificial chimerism mixture samples Mika et al. found high correlation between the estimated and the observed percentage values for two discriminating markers. The standard deviation of the four-time repeated measurements of a dilution series with one discriminating marker ranged from 1.8% to 3.7%. Comparing dPCR to the ‘gold-standard’ STR by fragment analyzes indicated good agreement between these two techniques in the detection range of 83–100% of donor chimerism [49]. The limit of detection of dPCR was estimated to be as low as 0.008% allowing the reliable determination of microchimerism. Additionally, assay precision was acceptable also in ranges of microchimerism, with a variation coefficient of 16% at 1:999 dilution [36]. The dPCR technique proved to be more sensitive in detection of relapse after allo-HSCT compared to qPCR [35]. Similarly to allo-HSCT, dPCR has also been widely used in the context of SOT to detect dd-cfDNA in combination with DIPs [32,50], SNPs [3], copy number variations [51], or HLA-disparities [17,18,43,52].

### 3.3. Next Generation Sequencing (NGS)

Next generation sequencing (NGS) is a massively parallel or deep sequencing, where millions of small fragments of DNA are sequenced in parallel affording an unprecedentedly high sensitivity. Sequenced fragments are pieced together by mapping individual reads to the human reference genome by bioinformatic tools. Several different NGS platforms exist with different chemistries and techniques. NGS can be used to sequence individual genes, targeting the whole exome or even the entire genome [53]. This technique is more sensitive than fragment analysis (0.01% to 1% vs. >1%). Compared to STR by fragment analyzes, NGS showed an excellent correlation [54,55]. The unprecedented capacity of NGS allows the simultaneous typing of large marker sets, predominantly SNPs, which has frequently been used to perform dd-cfDNA detection in various SOT settings. The large number of SNP markers applied clearly gives an unprecedented opportunity to distinguish two genomes, even in cases of unavailable donor pre-transplant DNA sample or in cases with multiple numerical chromosomal abnormalities. The main advantage of NGS is that it is applicable for simultaneous determination of chimerism or microchimerism as well as multiple disease specific markers to detect measurable residual disease (MRD). However, these techniques require substantial equipment background, reagent cost, trained bioinformatics expertise, standardization, and currently they are not always characterized with sufficiently short turn-around time to readily influence clinical decision making. The described approaches belonged either to non-commercially designed systems [17,18,52] or to commercially developed platform with an unequivocal intent of routine clinical application [56,57,58].

## 4. Sample Types, Preanalytics, and Isolation of Cell Free DNA

The volume of blood required for cfDNA analysis is usually between 6 and 10 mL. Concentrations in paired plasma and serum samples have revealed significantly higher cfDNA concentrations in serum than in plasma [59]. This is due to leukocyte damage during clotting in the serum tube with a single cell containing about 6 pg of DNA and adding up to 65 ng of DNA per mL of blood. As a consequence, serum samples are not advised, instead plasma from anticoagulated (predominantly EDTA) blood sample is used [60]. The use of tubes containing preservatives (PAXgene cfDNA tubes (Qiagen PreAnalytiX GmbH, Düsseldorf, Germany), Roche/Ariosa cfDNA collection tubes (Roche Diagnostics GmbH, Rotkreuz, Switzerland) and Streck BCT tubes (Streck Inc., Omaha, NE, USA, see [61]) to prevent hemolysis and to reduce the degradation of cfDNA is becoming increasingly common practice allowing an extended time to blood processing [62]. Interestingly, in the case of timely (up to 12 h at room temperature) processing, there was no significant difference between cfDNA concentrations in samples shipped in EDTA collection tubes versus samples shipped in cell-free DNATM blood collection tubes [63,64]. Plasma preparation typically involves two rounds of centrifugation: a classical, low speed centrifugation at 1000–2000 × *g* followed by a high speed centrifugation (16,000× *g*) [65]. In cases of a single centrifugation step at 800 × *g*, cfDNA concentrations were contaminated by genomic DNA [66].

Two main types of cfDNA extraction systems have frequently been used: column-based systems and magnetic beads systems. Studies comparing cfDNA extraction methods and kits revealed substantial differences in total DNA yield [67]. cfDNA samples may withstand three freeze-thaw cycles and storage at −20 °C. Storage time has no influence on specific sequences or mutations in cfDNA, and mutations can be detected several years after freezing plasma samples, but cfDNA content is decreasing with storage time, so quantification or characterization of cfDNA fragmentation, is preferred within 3 months after the extraction procedure [68].

Quantitation of extracted cfDNA may be performed with several techniques such as fluoro-spectroscopy, Qubit (Thermo Fisher Scientific Waltham, MA, US) fluorometry and qPCR. In addition, quality control of isolated cfDNA can be performed by fragment analysis by capillary electrophoresis. Based on fragment size, the relative amount of nucleosome-protected cfDNA fragments with 140–160 base pairs can be compared to degradation-fragments with higher molecular weight DNA [69,70]. Single-tube multiplex digital droplet PCR assays have also been described as alternative approaches to measure amplifiable DNA concentrations and fragment size of small amounts of cfDNA [62,71].

## 5. Role of Microchimerism in Solid Organ Transplantation

In the context of solid organ transplantations (SOT), the meaningful use of the term microchimerism is only possible for cfDNA in the circulation since intact graft cells are unlikely to survive in blood. Appearance of low amounts (usually less than 0.1%) of donor hematopoietic (passenger) cells in the recipient’s circulation has been repeatedly observed after SOT and prompted speculations about a potential favorable role of these cells in graft acceptance. However, later clinical studies failed to find consistent associations and this cell based microchimerism also showed large inter-individual variations (see the excellent review by Eikmans et al. [1]).

After SOT, beside cells of donor origin, recently an increasing attention has turned towards dd-cfDNA since upon immune-mediated, apoptotic, or necrotic cell damage, its amount increases. This may then be translated to elevated levels of absolute amount or of relative proportions of dd-cfDNA. Such changes may serve as sensitive surrogate biomarkers to monitor the health of the transplanted organ (graft). This has outstanding significance as the overall frequency of graft related post-SOT complications—i.e., rejection—immunosuppressive treatment mediated toxicity or infection can reach 50% of all SOT cases [72]. For the timely alleviation of these complications (e.g., appropriate adjustments of immunosuppressive therapies), systematic utilization of appropriate biomarkers is essential. To this end, monitoring the health of the allograft by a diverse array of laboratory parameters as well as therapeutic drug monitoring of immunosuppressive drugs has traditionally been used to assist therapeutic decision making. In addition, monitoring and characterization of donor specific antibodies (DSA) have recently gained increasing attention as the major causative factor of antibody mediated rejection (ABMR). However, the direct translation of DSA-profile data into clinical decision making has been challenging. It has been reported that up to 80% of patients with DSA do not have ABMR and additional factors, such as type and strength of DSA, IgG subclass, and the ability to bind complement, are important further determinants [73]. Besides the above-mentioned, non-invasive monitoring options, graft biopsy procedures have traditionally represented the gold standard for graft-damage evaluation which are invasive and costly, limiting their use in clinical practice. A significant proportion of biopsies yield inadequate specimen and major complications also occur relatively frequently. Furthermore, biopsy results are often compromised by expert reader variance and can lead to delayed diagnosis of active rejection, after which irreversible organ damage may have occurred [74].

Monitoring of dd-cfDNA in plasma as a non-invasive indicator of graft damage after SOT was first suggested as early as in 1998 by the most outstanding expert of the field, Lo et al. [75] followed by several early studies focusing on SOT [76,77,78,79]. At this stage, the techniques applied did not readily allow the accurate detection of dd-cfDNA below 1% corresponding to true microchimerism. In addition, restricted availability of suitable markers for donor vs. recipient distinction also hampered cohort studies of larger consecutive series. With dramatic technical advancement (see Section 2 and Section 3) both, the sensitivity and the informative marker availability problem has been solved allowing the completion of a number of cohort studies in various SOT settings. Studies on one hand attempted to characterize the precise behavior of dd-cfDNA in the early phase of SOT as well as to establish reference ranges documenting a sharp decline in the initial days and a proportion typically between 0.3% and 1.2% of total cfDNA in stable kidney transplant patients [16,19]. While for liver and transplants these proportion values are somewhat higher [16]. On the other hand, the majority of studies addressed the more exciting question namely how dd-cfDNA serves as a rejection biomarker. Several reviews have summarized these studies [72,80,81,82,83]. Out of these, in their recent systematic review, Knight et al. [84] published a large collection of relevant literature searches. However, no consistent distinction has been made between conference abstracts and peer-reviewed in extenso publications making it difficult to realize the number and extent of truly independent cohort studies. In addition, potential patient overlaps between cohorts are also impossible to identify in this manner.

Thus, we performed a focused literature overview of exclusively peer-reviewed papers addressing the potential role of plasma dd-cfDNA as a potential rejection marker in various SOT settings with an inclusion criteria of total cohort size equaling or exceeding *n* = 50 for kidney or *n* = 20 for other solid organs respectively. As shown in Table 2, a total of 15 studies met these criteria.

As an overall conclusion, significantly positive role was exclusively found across all organs for plasma dd-cfDNA as allograft injury biomarker, no study was found concluding the lack of association. In kidney transplant, the paramount prospective trial of Bloom et al. called “Circulating Donor-Derived Cell-Free DNA in Blood for Diagnosing Active Rejection in Kidney Transplant Recipients” (DART) had the strongest impact with subsequent positive decision about health insurance reimbursement in the US. By using a cut-off value of 1.0% of dd-cfDNA these authors found that this biomarker had stronger discriminating power between ABMR and absence of ABMR compared to cell mediated rejection versus lack that of [56]. The biopsy-linked patient selection design allowed the enrichment of patients with documented rejection episodes. Using essentially the same cohort additional aspects were also examined by the same center such as the joint diagnostic characteristics of dd-cfDNA and DSA [58] as well as biomarker performance details of dd-cfDNA with partial overlap with the original study [57]. The relevant UCSF and Australian studies indeed represent independent confirmations, albeit in smaller cohorts [18,51].

Heart transplant could be viewed as a pioneer of the field with an early study of a sizeable cohort from Stanford taking advantage of a large biorepository also containing plasma, allowing the selection more than a 100 sex-mismatched heart transplant cases [78] later confirmed in an independent prospective cohort using the novel NGS-based technique [85]. Among the further confirmatory studies a diagnostic method oriented study can be found by Grskovic et al. [17] as well as a pediatric heart transplant cohort examined with proprietary diagnostic [86] and a Spanish study using an alternative, technically less challenging diagnostic approach [32]. Regarding liver TX, besides an early case report [91] and small prospective cohorts [50,92], a larger (*n* = 115) multicentric study demonstrated significantly elevated dd-cfDNA levels in acute rejection compared to transplanted patients in stable phase or those with hepatitis C infection [87]. Similarly, in the field of lung TX, besides a few smaller studies, only the Stanford group was able to collect larger cohort after the initial study [85] with a multicentric prospective trial indicating markedly elevated dd-cfDNA prior to graft-rejection proven by biopsy [89] supplemented by a further study on essentially the same cohort [90].

Besides plasma, alternative test materials have also been used to monitor cfDNA after SOT. Sigdel et al. observed rather large inter-patient variations of cfDNA in urine samples of a sizeable (*n* = 61) kidney transplanted cohort with limited potential as a general diagnostic biomarker [93]. cfDNA was also measured in broncho-alveolar lavage (BAL) fluid after lung TX among 60 patients along with the chemokine CXCL10 and the combination of these markers significantly predicted allograft survival [52].

The most important limitation of plasma dd-cfDNA monitoring is its lacking specificity for rejection subtype, or specific histopathological alteration. However, due to its non-invasive character, the potentially rapid laboratory turn-around time, the increasing accessibility and the continuously decreasing cost, this approach may represent an early contributing diagnostic tool supplementing available classical indicators.

## 6. Role of Microchimerism in Allogeneic Hematopoietic Stem Cell Transplantation

### 6.1. General Applications of Chimerism Monitoring in Allo-HSCT

Chimerism analysis (detection of the relative ratio of donor and recipient hematopoiesis) from peripheral blood or bone marrow aspirates is a standard diagnostic procedure following allo-HSCT. The replacement of recipient cells by those of the donor called complete donor chimerism is an evidence for the successful engraftment and the reconstitution of hematopoiesis after allo-HSCT. After myeloablative conditioning, residual recipient hematopoiesis may indicate graft failure and/or persisting disease. Remaining or re-emerging recipient cells in the circulation, called mixed chimerism, after transplantation is mostly associated with relapse in cases of hematopoietic malignancies, although long term mixed chimerism without relapse has been described [94]. Persisting mixed chimerism is reported to reduce the graft versus leukemia (GvL) effect of alloreactive donor T cells [95,96,97]. In case of non-malignant disorders treated with non-myeloablative regimens, increasing mixed chimerism is an indicator of graft rejection.

The dynamics of serial chimerism monitoring facilitates therapeutic interventions such as modulation of immunosuppression, donor lymphocyte infusions, chemotherapy, or second transplantation both in myeloablative and in non-myeloablative settings [95]. Early, individualized interventions have the potential to balance between competing risks of graft failure, recurring disease, and the potentially life-threatening graft versus host disease (GvHD).

Analysis of STRs by fragment analysis (FA) is the most widely accepted choice of method for post-HSCT monitoring. Detailed recommendations are available for proper laboratory performance of STR chimerism monitoring [9]. The major disadvantage of STR chimerism monitoring is its low sensitivity (limit of detection: 1–5%). To circumvent this limitation, frequent monitoring (even weekly before day 200 after allo-HSCT in pediatric acute lymphoblastic leukemia), performing the analysis in subpopulations for the detection of lineage specific split chimerism are recommended, as acute leukemia relapse can occur within a relatively short time frame [98].

Chimerism monitoring provides a surrogate biomarker for tumor-specific measurable residual disease (MRD). Chimerism testing is suggested to be applied in combination with MRD monitoring in malignant hematopoietic diseases [99,100]. In several clinical settings, MRD assay is not available (due to the lack of identified disease specific somatic mutation) or clonal heterogeneity, in these cases chimerism testing remains the only follow-up marker after allo-HSCT.

### 6.2. Relevance of Microchimerism Monitoring in Allo-HSCT

In recent years, the sensitive detection of MRD (reaching at least 0.1% or deeper limit of detection) gained increasing attention, as patients with MRD-positive morphologic remission showed similar outcome to patients with active disease [99,101]. Introducing sensitive techniques like qPCR or dPCR enabled the reliable detection of microchimerism, which is defined as the presence of recipient cells below 1% in the circulation. Initially, this sensitivity was not a requirement for chimerism detection [102]. Earlier, the clinical utility of microchimerism detection after allo-HSCT was controversial and limited [39]. Low levels of undulating microchimerism might have been detected for years after allo-HSCT, which may not have been necessarily related to relapse. Studies focusing on the potential advantages and the clinical utility of sensitive chimerism detection are summarized in Table 3.

Several papers indicated that the dynamics of microchimerism was more closely correlated with relapse in acute leukemia. The reappearance of recipient DNA after complete donor chimerism is a warning sign, and increasing recipient chimerism in two consecutive samples reliably forecast relapse [28,103]. Stable or decreasing microchimerism levels might not indicate relapse. In acute leukemia, several lines of evidence regarding allo-HSCT chimerism monitoring suggest that sensitive methods (e.g., qPCR, dPCR) are superior compared to classic STR-fragment analysis in the prediction of hematological relapses: (i) the ratio of detected/all relapses for DIP/qPCR was 70–100% vs. 13–44% for STR/fragment analysis; (ii) the time difference in favor of DIP/qPCR vs. STR/fragment analysis was 26–90 days [13,27,34,44,96,104,105]. At the same time, the lack of recipient genotype in highly-sensitive chimerism analysis excludes the possibility of relapses with high certainty [28,103]. Systemic infections, especially virus infections, and acute GvHD were also variably associated with a slight increase in host microchimerism. Leukemia relapse is usually associated with a rapid expansion of host hematopoiesis [34,103,106].

Dynamics of chimerism also influence survival: complete donor and decreasing mixed chimerism present favorably, while increasing and stable mixed chimerism have adverse prognostic significance [105,107]. Recent studies indicated that not mixed chimerism, but also microchimerism after allo-HSCT show adverse overall survival compared to complete donor chimerism (mixed chimerism *p* < 0.0002; microchimerism *p* = 0.0201). The introduction of sensitive chimerism monitoring allows early detection of recipient hematopoiesis, early intervention, and the decrease of patients proportions with mixed chimerism above 1% (23% before and 15% after the introduction of sensitive chimerism test) [94].

Mixed chimerism, and also microchimerism, were found to strongly correlate with MRD-monitoring inasmuch, both biomarkers show similar time-course, when analyzed from the same sample type [29,96,100]. As a conclusion, chimerism including microchimerism monitoring is a reliable indicator of incipient acute myeloid leukemia (AML) relapse, especially in patients where no other specific molecular marker is available, which might affect up to 50% of allo-HSCT cases [34]. In case of chimerism monitoring, the lack of identifiable marker or subclonal heterogeneity do not alter the results however, cytogenetic alterations in the malignant clone resulting in the loss of the recipient allele carrying the respective marker may cause a false negative chimerism result.

### 6.3. Special Applications of Microchimerism Monitoring in Cellular Therapies

In hematology practice, special cellular therapies are becoming more and more frequent treatment options: examples are ‘microtransplantation’ in elderly AML patients, natural killer (NK) cell infusion or third-party donor antigen-specific T-cell infusions (virus specific T cells, VST) [109]. As these allogeneic cellular therapies are not intended to achieve durable engraftment, they frequently result in microchimerism not detectable with standard STR-based monitoring.

In a randomized, multicenter study of elderly AML patients, induction chemotherapy in combination with mobilized HLA-mismatched donor cells without GvHD-prophylaxis improved response rates with a low rate of mixed or complete donor chimerism (5/185) and severe acute GvHD (2/185). Microchimerism was detectable in 31% (8/26) sex-mismatched, informative transplants by SRY-qPCR (with up to 10 months follow-up) [110]. Microtransplantation was found to be inferior to HLA-matched sibling donor transplantation in allo-HSCT eligible patients for intermediate/high-risk AML in complete remission [111], but a case report described its utility as salvage therapy in refractory AML [112]. Haploidentical natural killer cell therapy is a safe and feasible therapeutic option in pediatric relapsed or refractory leukemia [113]. Adoptive transfer of VST cells aim to restore T-cell immunity and cure allo-HSCT patients with refractory, life-threatening viral infections like cytomegalovirus, Epstein–Barr virus, or adenovirus [114]. Kliman et al. described an ultrasensitive dPCR method capable to detect donor microchimerism after cellular therapies such as microtransplantation or VST [36].

### 6.4. Cell-Free DNA Chimerism Following Allo-HSCT

Plasma cfDNA markers as diagnostic applications have been intensively studied in the context of prenatal screening, solid tumors and SOT. The clinical usefulness of chimerism testing from plasma cfDNA in allo-HSCT has been sparsely reported. Initially, sex-mismatched allo-HSCT patients were screened to reveal the main cellular origin of plasma circulating cfDNA. Y-chromosome specific cfDNA was detected in plasma with a median of 6.9% in male patients with female donors, indicating the hematopoietic compartment as the main origin of plasma cfDNA [2].

Plasma cfDNA was found to be more sensitive than cellular chimerism in detecting relapse in leukemia patients with complete donor chimerism in polymorphonuclear cells. All patients with clinical relapse (16/84, 19%) had more than 10% of recipient cfDNA in the plasma. Altogether 60% of patients displayed various levels of mixed chimerism in plasma, including an additional 16 patients with >10% mixed chimerism level and without clinical relapse [115].

A further study indicated that mixed chimerism was detectable in cfDNA in a higher percentage of samples than in peripheral blood cells after allo-HSCT. Interestingly, plasma cfDNA-based microchimerism was capable of detecting isolated extramedullar relapses (central nervous system, *n* = 3), when in peripheral blood cells complete donor chimerism was observed [116]. Recipient derived cfDNA was elevated not only in relapse, but also in transplant related complications, especially in acute GvHD (aGvHD). The improvement of aGvHD symptoms during therapy coincided with the decrease in recipient cfDNA, in contrast, the lack of aGvHD amelioration was associated with stable or increasing levels of recipient cfDNA. This association suggested that recipient cell disruption in GvHD target organs can be a source of cfDNA. No correlation was found between mean recipient cfDNA percentage and aGvHD severity (grade I–II compared to grade III–IV), but organ specific involvements have not been described. Chronic GvHD (cGvHD) did not show an association with plasma cfDNA-based mixed chimerism in affected patients [116]. Plasma cell free mitochondrial DNA (mtDNA) was reported to be elevated in allo-HSCT patients. As an endogenous inflammatory signal, cell free mtDNA may activate a B-cell subset responsible for extensive cGvHD: the onset of cGvHD was accompanied with significantly elevated levels of plasma cell free mtDNA in patients with cGvHD [117].

## 7. Future Perspectives

In the context of SOT, additional independent, sufficiently large cohort studies are needed to further confirm the diagnostic value of microchimerism characterized by dd-cfDNA proportion in plasma. A further goal of these studies can be the targeting patients with subclinical graft damage for which currently the only diagnostic tool is the invasive organ biopsy. Applicability of dd-cfDNA based microchimerism detection as valuable information in the clinical decision-making process regarding actual immunosuppression should also be tested in a prospective setting. Routine application and comparisons of alternative detection techniques may also be attractive for future research since a considerable number of transplantation diagnostic facilities may not have access to the most advanced NGS-based determinations.

Although it is not widely accepted yet, monitoring of recipient cfDNA dynamics after allo-HSCT may be a valuable diagnostic tool to detect relapses at earlier time points, with special emphasis on extramedullary sites. Additionally, it can be utilized as a non-invasive biomarker for aGvHD activity. Further studies involving larger patient cohorts, as well as applying more sensitive methods than STR are necessary to address existing uncertainties regarding cfDNA chimerism testing in the allo-HSCT field.

## Figures and Tables

**Table 1:** Comparison of chimerism/microchimerism laboratory detection techniques.

Technique	Fragment Analysis^#^	qPCR	dPCR	NGS
Targeted Genetic Variant	limited number of multiallelic markers (STR)	limited number of biallelic markers (SNP, DIP)	limited number of biallelic markers (SNP, DIP)	unlimited number of biallelic markers (SNP)
Limit of Detection	>1%	1–0.01%	1–0.01%	1–0.01%
TAT	short	shortest	short	longer
Equipment Cost	considerable	relatively lower	considerable	considerable
Allo-HSCT Marker number*	3	2	2	not relevant
Advantages	gold standard ^#^; widespread application; large experience	high sensitivity; short TAT	high sensitivity; high precision	high number. of SNPs; simultaneous chimerism & MRD
Technical Limitations	stutter peak; preferential amplification; semi-quantitative	labor-intensive optimization; calibration curve; PCR inhibitors; duplicate low no. of variants	dependent on DNA concentration; low number of variants	infrastructure costs; longer TAT; bioinformatics; lack of standardization

^#^ Fragment analysis has been used for the longest time albeit due to its low sensitivity it is not suitable for microchimerism detection. * Minimum number of markers for allo-HSCT follow-up. Abbreviations: dPCR: digital PCR; DIP: deletion insertion polymorphism; HSCT: hematopoietic stem cell transplantation; MRD: measurable residual disease; NGS: next generation sequencing; qPCR: real time quantitative PCR; SNP: single nucleotide polymorphism; STR: short tandem repeat; TAT: turnaround time.

**Table 2:** Selected cohort studies focusing on the potential role of dd-cfDNA in SOT of various organs.

ID	Organ	Year	Author	Center/Country	Study Type	Study Design*	Total (*n*)	Rejection (*n*)
1	kidney	2017	Bloom [56]	Cedars-Sinai. LA, USA	prosp	biopsy	102	27
2	kidney	2018	Jordan [58]	Cedars-Sinai. LA, USA	retro	biopsy	87	16
3	kidney	2018	Sigdel [18]	UCSF, SF, USA	retro	consec	178	38
4	kidney	2019	Huang [57]	Cedars-Sinai LA, USA	prosp	biopsy	63	22
5	kidney	2019	Whitlam [51]	Melbourne, Australia	prosp	consec	55	13
6	heart	2011	Snyder [78]	Stanford, USA	retro	consec	112	12
7	heart	2014	DeVlaminck [85]	Stanford, USA	prosp	consec	65	n/a
8	heart	2016	Grskovic [17]	CareDX, Brisb., USA	retro	consec	101	27
9	heart	2018	Ragalie [86]	Wisconsin, USA	retro	biopsy	88	16
10	heart	2019	Macher [32]	Sevilla, Spain	prosp	consec	30	13
11	liver	2017	Schütz [87]	Gottingen, Germany	prosp	consec	107	17
12	liver	2019	Goh [50]	Melbourne, Australia	prosp	consec	20	3
13	lung	2015	DeVlaminck [88]	Stanford, USA	prosp	consec	51	n/a
14	lung	2018	Agbor-Enoh [89]	Stanford, NIH, USA	prosp	consec	157	34
15	lung	2019	Agbor-Enoh [90]	Stanford, NIH, USA	prosp	consec	106	n/a

* consecutive (consec): transplanted patients are consecutively included regardless of rejection; biopsy = biopsy-linked: sampling parallels biopsy indicated by organ-failure. Legend: Original studies were included if the cohort size equaled or exceeded *n* = 50 for kidney or *n* = 20 for other solid organs. Studies are listed according to organ transplanted then according to year published. Abbreviations: n/a: not available; prosp: prospective; retro: retrospective.

**Table 3:** Potential advantages and clinical applicability of sensitive microchimerism detection in allo-HSCT based selected papers.

First Author	Year	Country	*n*	Marker *	Clinical Utility of Microchimerism Detection
Jiménez- Velasco [13]	2005	Spain	61	VNTR, DIP	qPCR superior to FA in relapse prediction: 88% vs. 44%, 20 days prior FA; rising host chimerism kinetics in relapse
Koldehoff [105]	2006	Germany	269	STR, SNP, FISH XYSRY	qPCR superior to PAGE in relapse prediction: 90 days earlier; adverse overall survival in rising kinetics; SRY qPCR superior to XY FISH in relapse prediction: 86% vs. 28%, 143 days prior relapse
Wiedermann [107]	2010	Germany	75	DIP, SRY	rising host chimerism kinetics in relapse
Chen [27]	2011	Taiwan	126	STR, DIP	qPCR superior to FA in relapse prediction
Horky [96]	2011	Czech	46	STR, DIP	qPCR superior to FA in relapse prediction: 87% vs. 39%, 26 days prior FA.
Bach [44]	2015	Germany	16	STR, DIP	qPCR superior to FA in relapse prediction: 95 days earlier
Jacque [28]	2015	France	85	DIP	stable qPCR complete chimerism = negative predictor for relapse; rising host chimerism kinetics in relapse
Vicente [106]	2016	Brazil	41	DIP	early rising host chimerism kinetics in relapse
Ahci [34]	2017	Germany	30	STR, DIP	qPCR superior to FA in relapse prediction: 100% vs. 38%, 6 months prior relapse; rising or sustained host chimerism kinetics in relapse
Waterhouse [108]	2017	Germany	155	STR, SRY	dPCR superior to FA in relapse prediction: 90 days earlier
Cechova [94]	2018	Czech	474	STR, DIP	adverse overall survival in microchimerism compared to complete donor chimerism
Sellmann [103]	2018	Germany	71	DIP	stable qPCR complete chimerism = negative predictor for relapse; rising host chimerism positive predictor for relapse
Waterhouse [29]	2018	Germany	70	DIP, MRD	similar kinetics for chimerism and MRD
Tyler [37]	2019	USA	230	STR, DIP	combined STR-FA and DIP-qPCR in diagnostic algorithm; qPCR is more sensitive than FA
Valero-Garcia [35]	2019	Spain	28	DIP	dPCR superior to qPCR in relapse prediction

* STR, VNTR markers were tested with FA or PAGE; DIP, SNP, and SRY were tested with qPCR or dPCR. Legend: Original in extenso studies were included if clinical outcome of allo-HSCT (relapse, survival measures) was indicated in connection with chimerism levels below 1%. Studies are listed according to year published. Abbreviations: DIP: deletion-insertion polymorphism; FA: fragment analysis; FISH: fluorescence in situ hybridization; MRD: measurable residual disease; PAGE: poly-acrilamid gel-electrophoresis; qPCR: quantitative polymerase chain reaction; SNP: single nucleotide polymorphism; SRY: sex determining region Y; STR: short tandem repeat; VNTR: variable number tandem repeat.
